# Markerless Lung Tumor Motion Tracking by Dynamic Decomposition of X-Ray Image Intensity

**DOI:** 10.1155/2013/340821

**Published:** 2013-12-08

**Authors:** Noriyasu Homma, Yoshihiro Takai, Haruna Endo, Kei Ichiji, Yuichiro Narita, Xiaoyong Zhang, Masao Sakai, Makoto Osanai, Makoto Abe, Norihiro Sugita, Makoto Yoshizawa

**Affiliations:** ^1^Tohoku University Graduate School of Medicine, Sendai 980-8575, Japan; ^2^Hirosaki University Graduate School of Medicine, Hirosaki 036-8562, Japan; ^3^St. Luke's International Hospital, Tokyo 104-8560, Japan; ^4^Graduate School of Engineering, Tohoku University, Sendai 980-8579, Japan; ^5^Center for Information Technology in Education, Tohoku University, Sendai 980-8576, Japan; ^6^Cyberscience Center, Tohoku University, Sendai 980-8578, Japan

## Abstract

We propose a new markerless tracking technique of lung tumor motion by using an X-ray fluoroscopic image sequence for real-time image-guided radiation therapy (IGRT). A core innovation of the new technique is to extract a moving tumor intensity component from the fluoroscopic image intensity. The fluoroscopic intensity is the superimposition of intensity components of all the structures passed through by the X-ray. The tumor can then be extracted by decomposing the fluoroscopic intensity into the tumor intensity component and the others. The decomposition problem for more than two structures is ill posed, but it can be transformed into a well-posed one by temporally accumulating constraints that must be satisfied by the decomposed moving tumor component and the rest of the intensity components. The extracted tumor image can then be used to achieve accurate tumor motion tracking without implanted markers that are widely used in the current tracking techniques. The performance evaluation showed that the extraction error was sufficiently small and the extracted tumor tracking achieved a high and sufficient accuracy less than 1 mm for clinical datasets. These results clearly demonstrate the usefulness of the proposed method for markerless tumor motion tracking.

## 1. Introduction

In radiation therapy, to irradiate sufficient dose to tumors and avoid unnecessary dose to the surrounding healthy tissues are crucial to achieve significant treatment effects and reduce adverse effects. Stereotactic body radiation therapy (SBRT) can satisfy such clinical demand for accurate isocenter positioning to the static center of the target tumor volume [1]. Intrafractional tumor motion can, however, badly affect the accuracy of the irradiating position and additional margins should thus be designed to account for such geometric uncertainties [2, 3]. Inevitably, the larger margins cover the wider regions of surrounding healthy tissues. In this sense, motion management is necessary for effective treatment, especially for abdominal and thoracic tumors [2–4]. Indeed, such tumors can move several centimeter due to mostly respiratory and cardiac motions [5, 6].

To achieve highly accurate irradiation to moving tumors, tumor tracking to measure or monitor the motion can be an ideal direction. Image-guided techniques to capture the tumor motion [7–11] have thus been developed for the tumor tracking. A kV X-ray fluoroscopy is widely used for such image-guided radiation therapy (IGRT) because of its capability of direct position measurement of a target tumor inside the patient's body. However, image quality of the fluoroscopy may not be sufficient for accurate measurement of the tumor position, and thus fiducial gold markers, which form sufficient contrast to the surroundings on fluoroscopic images, are often implanted into or near the tumor [7, 12]. The markers position can be measured accurately and regarded as a good fiducial of the tumor position. Although implanted markers are very effective for the accurate monitoring, the implantation of markers is an additional burden in clinical routine [13]. Furthermore, for lung tumor cases, it is a serious problem that the implantation itself runs the risk of pneumothorax as high as 30% of such patients [14].

On the other hand, markerless techniques are fundamentally free from the risk and have thus been developed for “safer” tracking [15–20]. Among them, Berbeco et al. [15] and Mostafavi [16] developed a filter to enhance tumor contrast with the surrounding tissues by averaging tumor images at the same respiratory phase over several periods. This technique assumes that the geometric relation between the tumor and the other structures such as bones, blood vessels, and other tissues is the same for anytime at the same respiratory phase. However, the relation can change, and thus the technique often fails to measure the tumor position accurately. Meyer et al. [17] and Wilbert et al. [18] have evaluated a conventional template matching technique for tumor tracking on megavoltage portal images. They compared the tracking performance of the matching technique with several objective functions to be minimized. The technique is based on the nonfluoroscopic image assuming that target image intensities can directly be defined by observing the target itself and may not be affected by the other structures intensity. In contrast, the fluoroscopic intensities of pixels at which the tumor may be located are dependent on not only the tumor itself, but also the other structures passed through by the X-ray [21]. Due to the fluoroscopic characteristics, only insufficient measurements of the tumor motion can be achieved by such conventional techniques.

In this paper, we propose a new markerless technique for accurate measurement of the target position by decomposing the fluoroscopic pixel intensity, which is the sum of intensity components of the tumor and the other structures passed through by the X-ray, into the target intensity component and the others. In other words, the proposed technique can extract the intensity component of the target tumor from the fluoroscopic intensity. The extracted tumor intensity component is independent of the other intensity components and the extracted tumor has sufficient contrast with the surrounding area. Consequently, it can be used to measure the position accurately. The performance evaluation of the technique is conducted by using both phantom and clinical data.

The rest of the paper is organized as follows. In Section 2, a new method for extracting tumor intensity component is proposed for the markerless tracking. Performance analysis to evaluate the markerless technique is given in Section 3. Concluding remarks are described in Section 4.

## 2. Methods

### 2.1. Concept of Dynamic Decomposition

**Figure 1:**
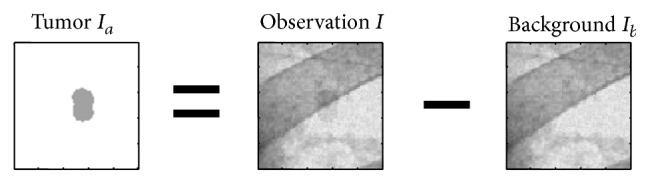
Concept of a tumor component extraction from an observed fluoroscopic image. Images are inversely colored and the tumor intensity is higher than the original for visibility.

Let us consider an *m* × *n* matrix *I* of a digital fluoroscopic image with pixel intensity *I*(*x*, *y*) at location (*x*, *y*), where *x* = 1,2,…, *n* and *y* = 1,2,…, *m*. The observed fluoroscopic intensity *I*(*x*, *y*) is the result of superimposition of a tumor intensity component *I*_*a*_(*x*, *y*) and the background intensity that is the sum of all the other components *I*_*b*_(*x*, *y*) of the rest of the structures passed through the X-rays. As shown in Figure 1, the extraction can then be represented by subtracting the background intensity *I*_*b*_ from the observed fluoroscopic one *I*

(1) $$\begin{array}{c} I_{a}\left(x,y\right)=I\left(x,y\right)-I_{b}\left(x,y\right). \end{array}$$

Note that the extraction of the tumor component *I*_*a*_ from the fluoroscopic image *I* is generally ill posed. In fact, (1) is an indefinite equation because not only the tumor image *I*_*a*_ but also the background *I*_*b*_ is unknown. Thus, (1) does not have a unique solution of the tumor image *I*_*a*_. Indeed, it is often very difficult for one to recognize a tumor in an X-ray fluoroscopic image. On the other hand, radiotherapists and radiologists can recognize the shape and image intensity component of the *moving* tumor on a fluoroscopic image sequence. This suggests that there is a mechanism to extract the shape and intensity component of the tumor not from each fluoroscopic image, but a sequence of them. In the following, we will formulate such extraction mechanism of the proposed *dynamic decomposition*.

It might be worth to mention that the spatial segmentation technique finds pixels or locations belonging to the target, while the intensity of a pixel may belong to more than two structures in a fluoroscopic image. Decomposition aims to extract the intensity component of the target structure from the observed fluoroscopic intensity. This is a main difference between the conventional segmentation and the proposed dynamic decomposition for tumor extraction.

Let us suppose that, for simplicity, tumor or background motion is a translation and consider each pixel of the extracted tumor component at a reference location (*x*_*r*_, *y*_*r*_) with intensity *I*_*a*_*r*__(*x*_*r*_, *y*_*r*_) that will move to a new location (*x*_*r*_ + *u*(*t*), *y*_*r*_ + *v*(*t*)) with intensity *I*_*a*_*r*__(*x*_*r*_ + *u*(*t*), *y*_*r*_ + *v*(*t*), *t*) at time *t*. In this case, the extracted tumor image at time *t* is written by the reference image *I*_*a*_*r*__ as

(2) $$\begin{array}{c} I_{a}\left(x,y,t\right)=I_{a_{r}}\left(x-u\left(t\right),y-v\left(t\right)\right), \end{array}$$

where (*u*(*t*), *v*(*t*)) denotes the displacement vector at time *t*.

Equation (2) implies that an extracted tumor image of any frame (time) of the image sequence, *I*_*a*_, can be represented by the reference *I*_*a*_*r*__ and the displacement vector (*u*(*t*), *v*(*t*)) at each time *t*. The tumor image extraction problem can then be solved by finding the reference image *I*_*a*_*r*__ common for all the frames and each displacement (*u*(*t*), *v*(*t*)).

For the background image at time *t* with intensity *I*_*b*_(*x*, *y*, *t*), the same condition using the reference image at the reference position (*x*_*b*_*r*__, *y*_*b*_*r*__) with intensity *I*_*b*_*r*__(*x*_*b*_*r*__, *y*_*b*_*r*__) can also be applied as

(3) $$\begin{array}{c} I_{b}\left(x,y,t\right)=I_{b_{r}}\left(x-u_{b}\left(t\right),y-v_{b}\left(t\right)\right), \end{array}$$

where (*u*_*b*_(*t*), *v*_*b*_(*t*)) denotes the background displacement. Similarly, the reference image *I*_*b*_*r*__ is common for any frames.

If the displacements are known, we may formulate simultaneous equations consisting of a set of ill-posed equations of (1) for several frames. Such simultaneous equations accumulate temporal image constrains that must be satisfied by the extracted tumor, the background, and the observed fluoroscopic intensities. Ideally, if the accumulated constrains are sufficient, the simultaneous equations will be solved and the tumor image *I*_*a*_ can be extracted. However, the number of frames required for regularization of the simultaneous equations is depended on the tumor and background motions and the other image configuration, and thus it is unknown in general. Then, we estimate the tumor image and optimize the estimation recursively, instead of solving the equations explicitly. In addition, the displacements are unknown in general, and thus we need to estimate them as well.

### 2.2. Dynamic Decomposition

#### 2.2.1. Displacement Estimation

The displacement of the tumor (*u*, *v*) in (2) can be measured by using a template matching technique [17]. Obviously, an ideal template for the tumor is the extracted tumor reference *I*_*a*_*r*__ that is unknown. Here the current estimation of the reference ${\hat{I}}_{a_{r}}$ can be used as an estimation of the template. Then, the displacement (*u*, *v*) can be estimated by matching the template ${\hat{I}}_{a_{r}}$ with the tumor image estimate ${\hat{I}}_{a}$.

From (1), an estimation of extracted tumor intensity matrix at time *t* can be given by subtracting the current estimate of the background image matrix from the fluoroscopic image matrix as

(4) I^a(t)=I(t)−I^b(t)+e(t),

(5) ≈I(t)−I^b(t),

where *e*(*t*) denotes the estimation error matrix at time *t* and

(6) I^a(t)≡I^a(x,y,t)=I^ar(x−u^(t),y−v^(t)),I^b(t)≡I^b(x,y,t)=I^br(x−u^b(t),y−v^b(t)).

The estimate of the background displacement $\left({\hat{u}}_{b}(t),{\hat{v}}_{b}(t)\right)$ can be given by matching the current background reference ${\hat{I}}_{b_{r}}$ with the fluoroscopic image *I*(*t*). This matching would be more accurate if the size of region of interest (ROI) is sufficiently large compared with the tumor size for ignoring a tumor effect on this background matching.

#### 2.2.2. Estimation of Tumor Intensity Component

A quadratic objective function to be minimized at time *t*, *J*(*t*), is given as

(7) $$\begin{array}{c} J\left(t\right)=\frac{1}{2}\mathrm{tr}\left(e^{\prime }\left(t\right)e\left(t\right)\right), \end{array}$$

where tr(*A*) and *A*′ denote trace and transposition of a matrix *A*, respectively. For simplicity, a sequential steepest descent method will be formulated for finding the best estimation, but many optimization methods can be applied to minimize various objective functions.

In the steepest descent method, the change of the tumor reference estimation ${\hat{I}}_{a_{r}}$, $\Delta {\hat{I}}_{a_{r}}$, to minimize *J* is given as

(8) $$\begin{array}{c} \Delta {\hat{I}}_{a_{r}}=-\eta \frac{\partial J}{\partial {\hat{I}}_{a_{r}}}, \end{array}$$

where *η* is a step size of the optimization. The update of the variable is then represented as

(9) $$\begin{array}{c} {\hat{I}}_{a_{r}}\left(k+1\right)={\hat{I}}_{a_{r}}\left(k\right)+\Delta {\hat{I}}_{a_{r}}, \end{array}$$

where *k* is an iteration number. The background reference estimation can be updated by the same manner, but in this paper it is updated by using the updated estimate of the tumor image ${\hat{I}}_{a}\left(t\right)={\hat{I}}_{a_{r}}\left(x-\hat{u}\left(t\right),y-\hat{v}\left(t\right),k+1\right)$,

(10) $$\begin{array}{c} {\hat{I}}_{b_{r}}\left(x+{\hat{u}}_{b}\left(t\right),y+{\hat{v}}_{b}\left(t\right)\right)=I\left(t\right)-{\hat{I}}_{a}\left(t\right) \end{array}$$

so that the error is kept to be 0.

Finally, the algorithm is summarized as follows.

**Step 1 .**

Initialize ${\hat{I}}_{a_{r}}$ and ${\hat{I}}_{b_{r}}$ (see (12) and (13)); *t* = 1.

**Step 2 .**

Estimate the displacement (*u*_*b*_(*t*), *v*_*b*_(*t*)) by matching ${\hat{I}}_{b_{r}}$ with *I*(*t*) and then estimate (*u*(*t*), *v*(*t*)) by matching ${\hat{I}}_{a_{r}}$ with ${\hat{I}}_{a}(t)$ in (5); *k* = 1.

**Step 3 .**

Calculate the error *e*(*t*) in (4).

**Step 4 .**

Update ${\hat{I}}_{a_{r}}$ and ${\hat{I}}_{b_{r}}$ by (9) and (10), respectively (tumor intensity estimation).

**Step 5 .**

*k* ← *k* + 1. Go back to Step 3 if *k* ≤ *K*, where *K* ( = 2 in this paper) is the maximum number of optimizing iterations; otherwise go to next Step.

**Step 6 .**

Estimate the motion (*u*(*t*), *v*(*t*)) as the center of mass of the binarized image of ${\hat{I}}_{a_{r}}$ (see Figure 5).

**Step 7 .**

*t* ← *t* + 1. Go back to Step 2 if *t* ≤ *T*, where *T* is the maximum number of frames; otherwise stop.

## 3. Experimental Results

We have evaluated performance of the proposed method by using phantom and clinical data sets.

The extraction performance was evaluated only for the phantom data because the ground truth of the extracted tumor image for clinical data is unknown. On the other hand, if the extraction is accurate, then the tracking is also accurate. Thus, the tracking performance can be a good index of the extraction performance as well.

For phantom motion tracking evaluation, the following error of Euclidean distance *e*_*p*_ between the measured displacement $\left(\hat{u}(t),\hat{v}(t)\right)$ and the *ground truth*  (*u*(*t*), *v*(*t*)) is calculated by averaging the distances over *T* frames of the fluoroscopic image sequence:

(11) $$\begin{array}{c} e_{p}=\frac{1}{T}\overset{T}{\underset{t=1}{\sum }}\sqrt{{\left(\hat{u}\left(t\right)-u\left(t\right)\right)}^{2}+{\left(\hat{v}\left(t\right)-v\left(t\right)\right)}^{2}}. \end{array}$$

For clinical data evaluation, three radiologists and medical physicists manually contoured the tumor image, and then three centers of mass of the contoured image were averaged and used as the ground truth of the tumor position (*u*(*t*), *v*(*t*)). For comparison purpose, we will also show motion tracking results by the same template matching technique [17] without the proposed tumor image extraction.

### 3.1. Phantom Data Case

#### 3.1.1. Phantom Data

A chest phantom fluoroscopic image without any tumor was taken first, and it was fluoroscopically superimposed by a moving phantom tumor image, which created 100 frames of size 600 × 250 pixels with spatial resolution 0.26 mm/pixel. The phantom tumor image used in this experiment is shown as *Tumor* *I*_*a*_ (left) in Figure 1. Motion of fiducial markers implanted into a lung cancer patient was used as the phantom tumor motion, which was measured every 0.033 s by using the real-time tumor-tracking (RTRT) system at Hokkaido University Hospital [7].

**Figure 2:**
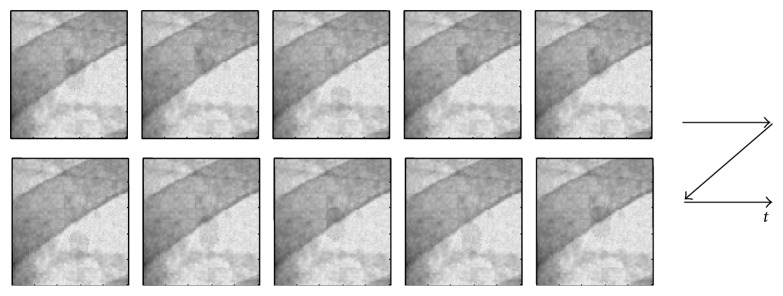
An example of the cropped phantom image sequence.

Figure 2 shows ten phantom frames, *I*, randomly chosen from the total hundred frames. The images shown are cropped to 100 × 100 pixels around the center of the original size. The left-upper image in Figure 2 shows the initial phantom fluoroscopic image, and the tumor location in the initial image is considered as the reference position with zero displacement (*u*, *v*) = (0,0).

#### 3.1.2. Tumor Image Extraction

In this experiment, the tumor outline was initialized manually on the first frame *I*(1), the left-upper image in Figure 2. Intensities inside the outline were initialized as a constant value *I*_0_ = 1. Then the reference image ${\hat{I}}_{a_{r}}$ was initialized as

(12) I^ar(x,y)={I0,if  (x,y)∈D00,otherwise,

where *D*_0_ denotes the region inside the initial outline. The background reference ${\hat{I}}_{b_{r}}$ was initialized by using the initial observation of the fluoroscopy subtracted by the initial tumor image as

(13) $$\begin{array}{c} {\hat{I}}_{b_{r}}=I-{\hat{I}}_{a_{r}}. \end{array}$$

**Figure 3:**
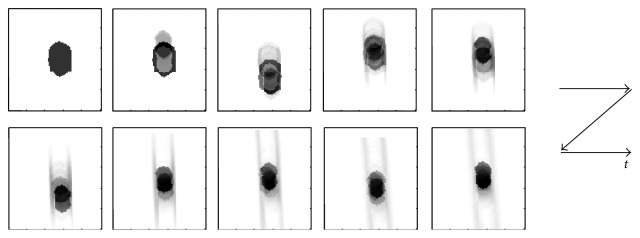
The cropped tumor image sequence during extraction for phantom case. The bigger shape of the initial tumor approaches gradually to the truth shown in Figure 1 (left).

**Figure 4:**
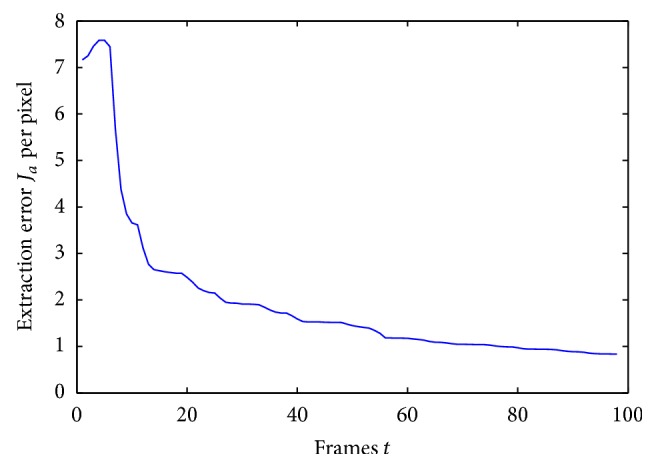
The extraction error *J*_*a*_ per pixel inside the cropped image as a function of iterations.

Figure 3 shows extracted images of the moving phantom tumor, ${\hat{I}}_{a}$, corresponding to the ten frames of phantom images in Figure 2. As seen in Figure 3, the extracted tumor image was initially different from the ground truth shown in Figure 1 (left), but approaching to the truth gradually. Indeed, an extraction error decreased gradually as shown in Figure 4. The error *J*_*a*_ is defined by using the error *e*_*a*_ between the extracted tumor image ${\hat{I}}_{a}$ and the ground truth *I*_*a*_ as

(14) $$\begin{array}{c} J_{a}\left(t\right)=\frac{1}{2}\mathrm{tr}\left(e_{a}^{\prime }\left(t\right)e_{a}\left(t\right)\right), \end{array}$$

where $e_{a}={\hat{I}}_{a}-I_{a}$.

**Figure 5:**
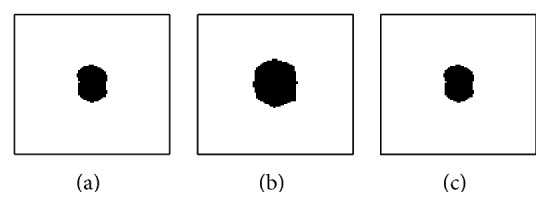
Binarized extraction results: (a) phantom tumor image (ground truth), (b) tumor image initialized manually, and (c) the final intensity component of the extracted tumor. The initial shape of the tumor is obviously bigger than the ground truth, but the final extraction of the tumor seems sufficiently similar to the truth.

Figure 5 shows a comparison between the truth and the extraction results. In this figure, images were binarized for visibility. The initial shape of the tumor shown in Figure 5(b) was obviously bigger than the truth in Figure 5(a). Nevertheless, the extracted tumor intensity component shown in Figure 5(c) seems sufficiently similar to the truth and the error *J*_*a*_, reflecting the extraction error of the different shape and 2 line motion traces seen in Figure 3, was negligible for accurate tumor tracking (see Section 3.1.3). We may thus conclude that the tumor image can be extracted from the X-ray fluoroscopic image sequence.

#### 3.1.3. Motion Tracking

By using the binarized extracted tumor, the average error of the motion measurement *e*_*p*_ in (11) converged to zero, implying that the tumor motion can be measured within the spatial resolution, for example, 0.26 mm/pixel in this example. On the other hand, the error was more than 2.0 mm by using the same matching technique [17] without the proposed tumor extraction due to the fluoroscopic characteristics especially mismatching with the background structure. The comparison clearly demonstrates effectiveness of tumor extraction from the fluoroscopic images.

### 3.2. Clinical Data Cases

We also applied the proposed method to three clinical datasets of fluoroscopic image sequences.

#### 3.2.1. Clinical Data

Fluoroscopic images of size 512 × 512 pixels with spatial resolution 0.42 mm/pixel were taken by the X-ray simulator system (Ximatron CX, Varian Medical Systems, Palo Alto, CA) at Tohoku University Hospital. The sampling interval of the image observation was every 0.5 s (i.e., 2 images/s). The number of image frames was 18 for each case. The less sampling frequency and smaller number of images make it more difficult for the proposed method to extract the tumor image because the number of accumulated constrains described in Section 2.1 is less than the phantom case. However, the number of frames can be larger or even equal to that of phantom case without clinical difficulty before the therapeutic fraction. The peak-to-peak displacements in craniocaudal direction were 9.42 mm, 5.88 mm, and 7.14 mm for cases 1, 2, and 3, respectively.

#### 3.2.2. Tumor Image Extraction

**Figure 6:**
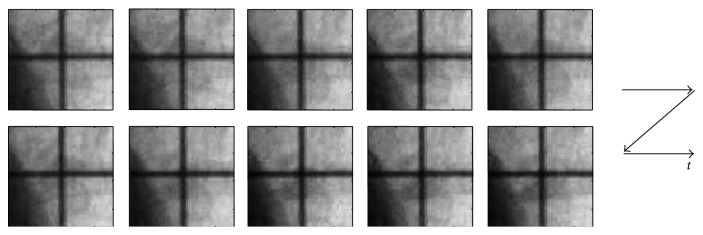
An example of the cropped image sequence of clinical Case 1.

**Figure 7:**
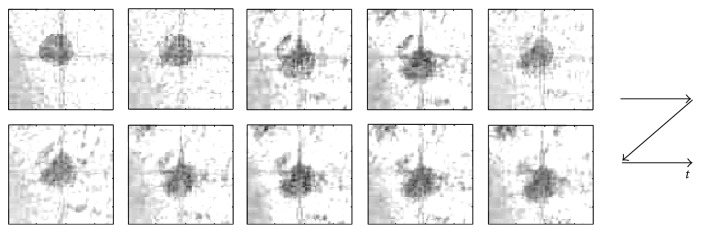
The cropped tumor image sequence during extraction for clinical Case 1. The final extraction seems similar to its unknown truth. The extracted tumor is clearer than the original fluoroscopy and thus useful for tracking.

Figures 6 and 7 show a fluoroscopic sequence of 10 frames chosen from tested 18 frames of the clinical case 1 and the corresponding frames of extracted tumor images, respectively. As seen in Figure 6, clinical images have different characteristics from the phantom case and can also be different from the model supposed in Section 2.1 These include unclearness of tumor contour, low contrast, noisiness, and changes of intensities possibly caused by motion of blood vessels, cardiac motion, and changes of exposure time. Although the characteristics may badly affect the extraction accuracy, such as blurred and noisy extraction, the result demonstrates that extracted tumors can be recognized clearer than those in the original fluoroscopic images.

#### 3.2.3. Motion Tracking

Table 1 summarizes tumor motion tracking performance for three clinical cases. First, by using the proposed extraction, the motion tracking errors for all cases are less than the conventional method without extraction. For example, the average with standard deviation of the tracking error for the three cases by the proposed method is 0.741 ± 0.230 mm and less than 1.721 ± 0.463 mm by the conventional method.

Second, the fact that the error is also less than a minimum clinical requirement within 1.0 mm may be more important. It might be worth to mention that even though the extraction image was blurred and noisy as shown in Figure 7, the method can still achieve a good tracking performance. These clearly demonstrate the clinical usefulness of the extraction for the markerless tumor motion tracking with sufficient accuracy.

### 3.3. Discussions

In the experiments, we manually initialized the tumor outline and intensities. Indeed, the radiologists can easily draw a rough outline on the fluoroscopy. Note that even started from a rough initial estimation of the outline and intensities, the proposed extraction can achieve a good tracking performance within 1 mm accuracy for both phantom and clinical cases. On the other hand, the more accurate initial estimation gives the more accurate extraction, especially for clinical cases. Such accurate outlines are available by using the X-ray CT image or digitally reconstructed radiography (DRR). Thus, the more accurate tracking can be achieved by the proposed extraction with the more accurate initial outline.

As mentioned earlier, better optimization techniques can improve extraction performance. The objective function *J*(*t*) of the sequential optimization formulated in this paper involves only one frame constrain and is good for real-time computation during treatment. On the other hand, constrains from more than 2 frames can simultaneously be incorporated into an objective function of a batch optimization, such as *J*_batch_ = ∑_t_ *J*(*t*). This is good for offline computation and can provide better intensity components before treatment. In this case, larger iterations *K* may be chosen. For further improvement, many tracking techniques other than the simple template matching [17] can also be incorporated into the proposed method. In fact, the phase only correlation [22–25] for low contrast cases and the particle filters [26–28] for noisy and stochastic deformation can be applied to the extracted tumor image.

Although the proposed technique can achieve real-time tracking, the current radiotherapy machine may have latency to control the irradiation position. In this case, motion prediction based on the real-time tracking can be used and such prediction methods have been proposed [4].

The number of clinical data used is not good enough and the image quality of clinical data is different from that of the phantom case, but this is because no new or extra data were taken other than from normal planning routine to avoid any extra radiation dose for the developmental phase. However, a large number of clinical data with the same image quality of the phantom case will be tested for the evaluation phase of the proposed method.

## 4. Conclusions

We have developed the dynamic decomposition method to extract the moving lung tumor image component from kV X-ray fluoroscopic images and applied it to the tumor tracking. The tracking does not require any fiducial markers implanted into the tumor and thus is fundamentally free from the risk of implantation troubles. Sufficiently high accuracy of the extraction and motion tracking has been demonstrated by using both phantom and clinical datasets. The results suggested that the proposed method is an ideal solution for the implantation risk and can achieve a low-risk and highly accurate tumor motion tracking for the real-time IGRT.

## Figures and Tables

**Table 1:** Motion tracking errors in millimeter for clinical cases.

Methods	Case 1	Case 2	Case 3	Average	Std. dev.
Without extraction	2.339	1.222	1.602	1.721	0.463
Proposed extraction	0.577	1.004	0.641	0.741	0.230

## References

[1] Dilling T. J., Hoffe S. E. (2008). Stereotactic body radiation therapy: transcending the conventional to improve outcomes. *Cancer Control*.

[2] Senan S., Chapet O., Lagerwaard F. J., Ten Haken R. K. (2004). Defining target volumes for non-small cell lung carcinoma. *Seminars in Radiation Oncology*.

[3] Senan S., de Ruysscher D., Giraud P., Mirimanoff R., Budach V. (2004). Literature-based recommendations for treatment planning and execution in high-dose radiotherapy for lung cancer. *Radiotherapy and Oncology*.

[4] Homma N., Sakai M., Endo H., Mitsuya M., Takai Y., Yoshizawa M. (2009). A new motion management method for lung tumor tracking radiation therapy. *WSEAS Transactions on Systems*.

[5] Murphy M. J. (2004). Tracking moving organs in real time. *Seminars in Radiation Oncology*.

[6] Stevens C. W., Munden R. F., Forster K. M. (2001). Respiratory-driven lung tumor motion is independent of tumor size, tumor location, and pulmonary function. *International Journal of Radiation Oncology Biology Physics*.

[7] Shirato H., Shimizu S., Shimizu T., Nishioka T., Miyasaka K. (1999). Real-time tumour-tracking radiotherapy. *The Lancet*.

[8] Takai Y., Mitsuya M., Nemoto K. (2001). Development of a new linear accelerator mounted with dual fluoroscopy using amorphous silicon flat panel X-ray sensors to detect a gold seed in a tumor at real treatment position. *International Journal of Radiation Oncology∗Biology∗Physics*.

[9] Berbeco R. I., Jiang S. B., Sharp G. C., Chen G. T. Y., Mostafavi H., Shirato H. (2004). Integrated radiotherapy imaging system (IRIS): design considerations of tumour tracking with linac gantry-mounted diagnostic x-ray systems with flat-panel detectors. *Physics in Medicine and Biology*.

[10] Britton K. R., Takai Y., Mitsuya M., Nemoto K., Ogawa Y., Yamada S. (2005). Evaluation of inter- and intrafraction organ motion during intensity modulated radiation therapy (IMRT) for localized prostate cancer measured by a newly developed on-board image-guided system. *Radiation Medicine*.

[11] Wiersma R. D., Mao W., Xing L. (2008). Combined kV and MV imaging for real-time tracking of implanted fiducial markers. *Medical Physics*.

[12] Keall P. J., Todor A. D., Vedam S. S. (2004). On the use of EPID-based implanted marker tracking for 4D radiotherapy. *Medical Physics*.

[13] Zhu X., Bourland J. D., Yuan Y. (2009). Tradeoffs of integrating real-time tracking into IGRT for prostate cancer treatment. *Physics in Medicine and Biology*.

[14] Onishi H., Hiraoka M. *Detailed Explanation of Extracranial Stereotactic Radiotherapy—Details of the Guidelines and Exposure Manual*.

[15] Berbeco R. I., Mostafavi H., Sharp G. C., Jiang S. B. (2005). Towards fluoroscopic respiratory gating for lung tumours without radiopaque markers. *Physics in Medicine and Biology*.

[16] Mostafavi H. Systems and Methods for Processing X-ray Images.

[17] Meyer J., Richter A., Baier K., Wilbert J., Guckenberger M., Flentje M. (2006). Tracking moving objects with megavoltage portal imaging: a feasibility study. *Medical Physics*.

[18] Wilbert J., Meyer J., Baier K. (2008). Tumor tracking and motion compensation with an adaptive tumor tracking system (ATTS): system description and prototype testing. *Medical Physics*.

[19] Cui Y., Dy J. G., Sharp G. C., Alexander B., Jiang S. B. (2007). Robust fluoroscopic respiratory gating for lung cancer radiotherapy without implanted fiducial markers. *Physics in Medicine and Biology*.

[20] Endo H., Homma N., Takai Y., Yoshizawa M. (2010). Simultaneous estimation of lung tumor image and intrafractional motion without implanted markers using kV-X-ray fluoroscopy for image-guided radiotherapy. *Medical Physics*.

[21] Okabe T., Fujita K. *Medical Imaging and Engineering*.

[22] de Castro E., Morandi C. (1987). Registration of translated and rotated images using finite fourier transforms. *IEEE Transactions on Pattern Analysis and Machine Intelligence*.

[23] Srinivasa Reddy B., Chatterji B. N. (1996). An FFT-based technique for translation, rotation, and scale-invariant image registration. *IEEE Transactions on Image Processing*.

[24] Takita K., Aoki T., Sasaki Y., Higuchi T., Kobayashi K. (2003). High-accuracy subpixel image registration based on phase-only correlation. *IEICE Transactions on Fundamentals of Electronics, Communications and Computer Sciences*.

[25] Zhang X., Abe M., Kawamata M. (2011). Reduction of computational cost of POC-based methods for displacement estimation in old film sequences. *IEICE Transactions on Fundamentals of Electronics, Communications and Computer Sciences*.

[26] Doucet A., Godsill S., Andrieu C. (2000). On sequential Monte Carlo sampling methods for Bayesian filtering. *Statistics and Computing*.

[27] Crisan D., Doucet A. (2002). A survey of convergence results on particle filtering methods for practitioners. *IEEE Transactions on Signal Processing*.

[28] Gordon N. J., Salmond D. J., Smith A. F. M. (1993). Novel approach to nonlinear/non-Gaussian Bayesian state estimation. *IEE Proceedings F*.

